# A Bounding Box-Based Radiomics Model for Detecting Occult Peritoneal Metastasis in Advanced Gastric Cancer: A Multicenter Study

**DOI:** 10.3389/fonc.2021.777760

**Published:** 2021-12-03

**Authors:** Dan Liu, Weihan Zhang, Fubi Hu, Pengxin Yu, Xiao Zhang, Hongkun Yin, Lanqing Yang, Xin Fang, Bin Song, Bing Wu, Jiankun Hu, Zixing Huang

**Affiliations:** ^1^ Department of Radiology, West China Hospital, Sichuan University, Chengdu, China; ^2^ Department of Gastrointestinal Surgery and Laboratory of Gastric Cancer, Collaborative Innovation Center for Biotherapy, State Key Laboratory of Biotherapy, West China Hospital, Sichuan University, Chengdu, China; ^3^ Department of Radiology, First Affiliated Hospital of Chengdu Medical College, Chengdu, China; ^4^ Institute of Advanced Research, Infervision, Beijing, China; ^5^ Department of Radiology, People’s Hospital of Leshan, Leshan, China

**Keywords:** gastric cancer, peritoneal metastasis, radiomics, bounding box, computed tomography

## Abstract

**Purpose:**

To develop a bounding box (BBOX)-based radiomics model for the preoperative diagnosis of occult peritoneal metastasis (OPM) in advanced gastric cancer (AGC) patients.

**Materials and Methods:**

599 AGC patients from 3 centers were retrospectively enrolled and were divided into training, validation, and testing cohorts. The minimum circumscribed rectangle of the ROIs for the largest tumor area (R_BBOX), the nonoverlapping area between the tumor and R_BBOX (peritumoral area; PERI) and the smallest rectangle that could completely contain the tumor determined by a radiologist (M_BBOX) were used as inputs to extract radiomic features. Multivariate logistic regression was used to construct a radiomics model to estimate the preoperative probability of OPM in AGC patients.

**Results:**

The M_BBOX model was not significantly different from R_BBOX in the validation cohort [AUC: M_BBOX model 0.871 (95% CI, 0.814–0.940) *vs.* R_BBOX model 0.873 (95% CI, 0.820–0.940); p = 0.937]. M_BBOX was selected as the final radiomics model because of its extremely low annotation cost and superior OPM discrimination performance (sensitivity of 85.7% and specificity of 82.8%) over the clinical model, and this radiomics model showed comparable diagnostic efficacy in the testing cohort.

**Conclusions:**

The BBOX-based radiomics could serve as a simpler reliable and powerful tool for the preoperative diagnosis of OPM in AGC patients. And M_BBOX-based radiomics is simpler and less time consuming.

## Introduction

Gastric cancer (GC) is one of the most common and deadly carcinomas in the world (1). Peritoneal metastasis (PM), one of the most common forms of metastasis in GC, occurs in ~53%-80% of GC patients with distant metastasis (2, 3) and is generally regarded as an incurable condition with a poor prognosis (4, 5). It was reported that the median survival time among PM patients is 3–6 months (5), and the treatment options are limited (6). New treatment strategies such as intraperitoneal chemotherapy and extensive intraoperative peritoneal lavage are associated with an improved prognosis for those patients (7–9). Therefore, noninvasive preoperative detection of PM of advanced gastric cancer (AGC) is crucial for avoiding unnecessary surgery and selecting optimal therapy in clinical practice.

CT is recommended as the first-line imaging modality for the detection of PM (10). However, PMs (<5 mm) are frequently missed on CT images (11). In 10 to 30% of patients with negative CT images, intraperitoneal metastases are found during either laparoscopy or surgical exploration (11, 12), called occult peritoneal metastases (OPMs).

Artificial intelligence, especially deep learning, has been explored in predicting PM in GC (13, 14), such as previous research from our team (14). However, the interpretation is under elucidated due to its nature of “black box”. Radiomics is an emerging field focusing on the high-dimensional mineable feature set captured from imaging data using a series of quantitative characteristic algorithms. The effective extraction and modeling of digital information is expected to aid in the assessment and differential diagnosis of gastric tumors (15–20). For OPMs in AGC, some studies (21–23) have utilized radiomics analysis of preoperative CT texture features to make a preoperative, noninvasive OPM diagnosis. All regions of interest (ROIs) were drawn manually in these studies. However, the manual annotation of many medical images is time consuming, expensive, and inefficient. In addition, most medical image annotations require a certain level of expertise, which can add additional work for the physician. Recently, we applied another annotation method—an easy-to-use, time-saving and inexpensive method referred to as the bounding box (BBOX)—that has been used in some clinical areas (24–28), such as segmentation, diagnosis, and classification, but has not yet been applied to the radiomics analysis of OPM in GC patients.

The BBOX, which contains both the tumor tissue and peritumoral area (PERI), covers more regions than manual annotation of the tumor alone. A few studies have revealed that the combined radiomic signature of intratumoral and peritumoral regions can provide valuable information for the prediction of the Lauren classification of GC (29), the pathological complete response to neoadjuvant chemoradiation of esophageal squamous cell carcinoma (30), and the outcomes and chemotherapy response in GC (31, 32).

Therefore, in this study, we hypothesized that ROI annotation by BBOX might provide adequate information for the diagnosis of OPM. We aimed to develop and validate a BBOX-based radiomics model for the preoperative, noninvasive diagnosis of OPM in AGC patients.

## Materials and Methods

**Figure 1:**
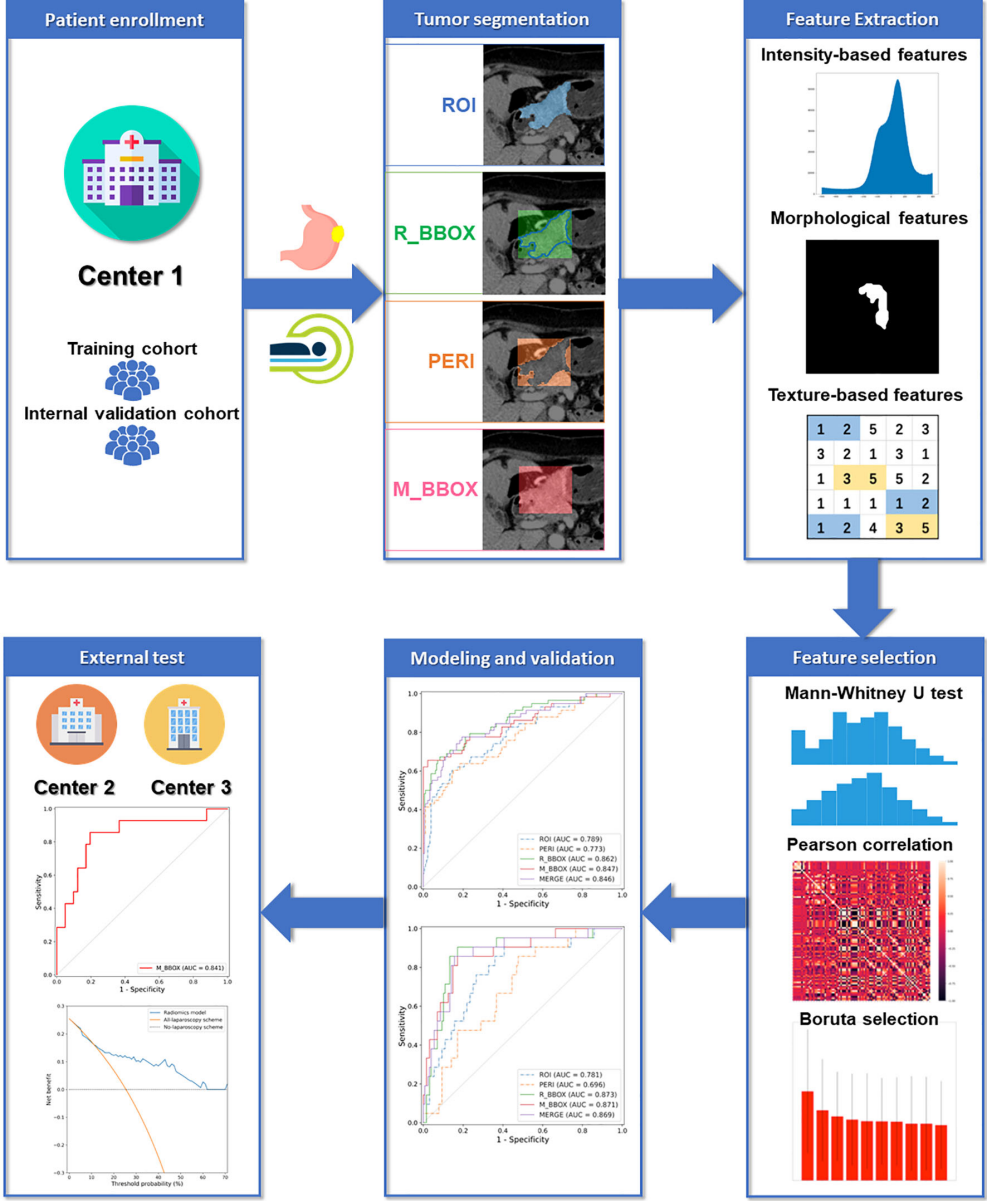
Radiomics processing flowchart.

The radiomics processing flowchart of this study is shown in **Figure 1**.

### Patients

This multicenter retrospective study was approved by the institutional review board of each center, and the informed consent requirement was waived. A total of 599 patients from three centers were divided into three cohorts: one training cohort (395 patients from center 1), one internal validation cohort (149 patients from center 1), and one external testing cohort (55 patients from center 2 and center 3). The details of PM status confirmation are described in **Section 1** in the **Supplementary Material**. The inclusion criteria and exclusion criteria are available in **Section 2** in the **Supplementary Material**.

### CT Image Acquisition and Data Preparation

The details of the CT protocol are presented in **Section 3** and **Table S1** in the **Supplementary Material**. Portal vein phase CT images were first exported to ITK-SNAP software (version 2.2.0; www.itksnap.org) for manual segmentation. GC lesions were then manually annotated by a radiologist with 5 years of experience in gastroenterology imaging and confirmed by another abdominal specialist with 14 years of experience.

**Figure 2:**
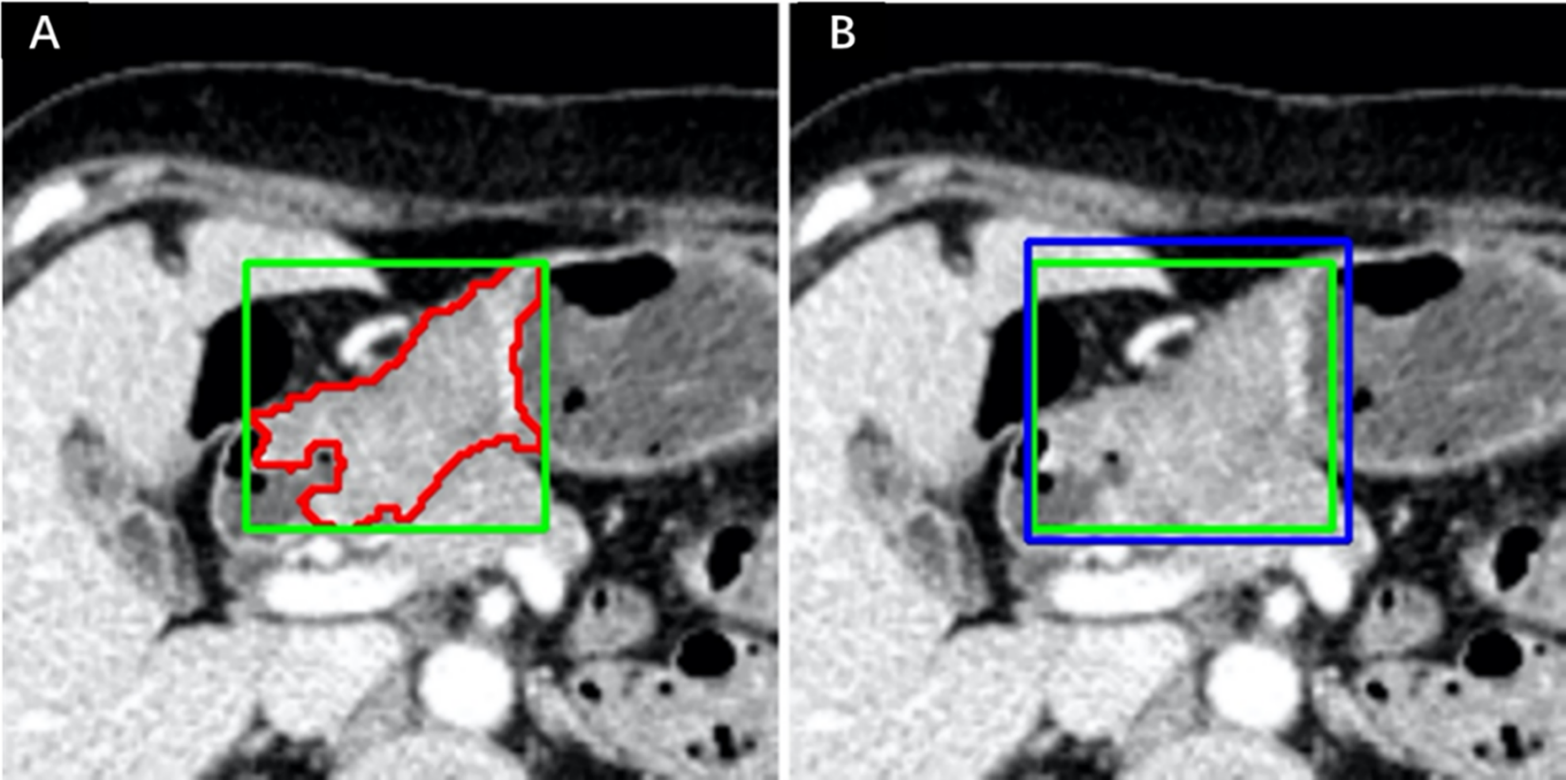
Annotation example in one patient. **(A)** ROI and R_BBOX indicated by the red and green lines, respectively. **(B)** M_BBOX indicated by the blue line; R_BBOX same as in **(A)**.

These two radiologists reviewed all slices obtained from each patient, selected the slice with the largest tumor area, and manually delineated the lesion to obtain the final ROIs. The BBOX was obtained by calculating the minimum circumscribed rectangle of the ROI, as shown in **Figure 2A**, called the R_BBOX. The nonoverlapping area between the ROI and BBOX was regarded as the PERI. For all patients, the radiologist was required to determine the smallest rectangle that could completely contain the tumor area and directly perform BBOX annotation. The manually obtained BBOX annotation was called M_BBOX. An example is shown in **Figure 2B**, as indicated by the blue line.

### Radiomic Feature Extraction

Radiomic features were extracted using Pyradiomics (33), an open-source radiomics toolbox. Eight classes of stable radiomic features were extracted according to the standards in the *Image Biomarker Standardization Initiative (* 34). Ultimately, 119 quantitative two-dimensional (2D) radiomics features were extracted for each annotation of the ROI, R_BBOX, PERI and M_BBOX. For each patient in the training cohort and validation cohort, we constructed an annotation type called MERGE, which includes the features of both the ROI and the PERI. Thus, the number of features in the MERGE region was 238. The MERGE approach is another way to analyze the tumor area and PERI at the same time.

### Feature Selection in Terms of Reproducibility

Two months after data annotation, 50 patients were randomly selected from the training cohort and reannotated by the previous radiologist. The same annotation rules were applied; that is, the ROIs and M_BBOXes of 50 patients were manually delineated. The R_BBOX and PERI for each patient could be obtained based on the ROI. For each patient, the radiomics features of the ROI, R_BBOX, PERI, M_BBOX and MERGE could be obtained. To evaluate the intraobserver agreement between the two repeated annotations, intraclass correlation coefficients (ICCs) were utilized. An ICC greater than 0.75 was considered a sign of reproducibility for the observer, and features with ICCs lower than 0.75 were excluded in the following feature selection process.

### Feature Selection and Classifier Modeling

As shown in **Section 4** in the **Supplement,** the feature selection process included three steps, with the aim of avoiding overfitting during the model-building process and potential biases associated with the results.

After the three-step feature selection process, the remaining radiomics features were used to construct a multivariate logistic regression model for OPM positivity prediction, namely, the radiomics model.

We used independent predictors of preoperative clinical characteristics to build a clinical model by multivariate logistic regression for comparison. The independent predictors were also screened by multivariate logistic regression analysis. Backward stepwise selection was performed based on the Akaike information criterion (AIC) (35).

### Four Sizes of Input Boxes

Four sizes of input boxes containing the primary tumor with different amounts of surrounding tissues were used to evaluate their diagnostic performance, as shown in **Section 6** in the **Supplement**.

### Statistical Analysis

Differences in continuous variables were analyzed with the Mann-Whitney U test, and differences in categorical variables were analyzed with the chi-squared test. The radiomic classification model and the clinical classification model were evaluated with the validation cohort. The variance in performance was assessed using receiver operating characteristic (ROC) curve analysis and quantified by assessing the areas under the ROC curves (AUCs) (36). The DeLong test (37, 38) was used for statistical comparisons of ROC curves. Considering the class imbalance in the validation cohort and testing cohort, bootstrapping (n = 1000) was used to calculate tfrhe 95% confidence interval (CI). A decision curve was plotted to evaluate model efficacy by quantifying the net benefits at different probability thresholds. All statistical analyses were performed with R software (version 3.5.0; http://www.Rproject.org) and SPSS 22.0 (IBM, Armonk, NY, USA). Differences with a two-tailed p-value less than 0.05 were considered statistically significant.

## Results

### Clinical Characteristics

The demographic information is shown in **Table 1**. **Table 1** shows significant differences between the lesion location and Borrmann type between OPM-positive and OPM-negative patients in the training cohort (p < 0.001). There were no significant differences in age, sex, carcinoembryonic antigen (CEA), or carbohydrate antigen 19–9 (CA19–9) between OPM-positive and OPM-negative patients in the entire cohort.

**Table 1:** Characteristics of the patients.

Characteristic	Training cohort (n = 395)			Validation cohort (n = 149)			Testing cohort (n = 55)		
	OPM-Pos (n = 58)	OPM-Neg (n = 337)	*p*	OPM-Pos (n = 21)	OPM-Neg (n = 128)	*p*	OPM-Pos (n = 14)	OPM-Neg (n = 41)	*p*
**Age (years, mean ± SD)**	58.17 ± 13.24	57.99 ± 11.74	0.451	55.86 ± 16.33	60.41 ± 10.76	0.121	60.61 ± 10.71	66.57 ± 12.34	0.122
**Sex (N, %)**			0.270			0.469			
Male	34 (58.6%)	226 (67.1%)		12 (57.1%)	87 (68.0%)		11 (78.6%)	26 (63.4%)	
Female	24 (41.4%)	111 (32.9%)		9 (42.9%)	41 (32.0%)		3 (21.4%)	15 (36.6%)	
**Location (N, %)**			0.002			0.116			0.917
U/U+M	12 (20.7%)	68 (20.2%)		3 (14.3%)	36 (28.1%)		2 (14.3%)	9 (22.0%)	
M/M+L	20 (34.5%)	63 (18.7%)		6 (28.6%)	18 (14.1%)		3 (21.4%)	8 (19.5%)	
L/L+D	18 (31.0%)	181 (53.7%)		9 (42.9%)	63 (49.2%)		8 (57.1%)	19 (46.3%)	
U+E	1 (1.7%)	11 (3.3%)		0 (0.0%)	5 (3.9%)		0 (0.0%)	3 (7.3%)	
Whole stomach	7 (12.1%)	14 (4.2%)		3 (14.3%)	6 (4.7%)		1 (7.1%)	2 (4.9%)	
**Borrmann type (N, %)**			0.000			0.009			0.123
Types 1, 2	48 (82.8%)	194 (57.6%)		18 (85.7%)	71 (55.5%)		1 (10.0%)	9 (39.1%)	
Types 3, 4	10 (17.2%)	143 (42.4%)		3 (14.3%)	57 (44.5%)		9 (90.0%)	14 (60.9%)	
Unknown	0	0		0	0		4	18	
**CEA (N, %)**			0.313			0.128			0.616
Normal	36 (62.1%)	235 (69.7%)		18 (85.7%)	88 (68.8%)		8 (80.0%)	28 (87.5%)	
Elevated	22 (37.9%)	102 (30.3%)		3 (14.3%)	40 (31.2%)		2 (20.0%)	4 (12.5%)	
Unknown	0	0		0	0		4	9	
**CA19–9 (N, %)**			0.091			0.221			1.000
Normal	37 (63.8%)	254 (75.4%)		11	88		8 (80.0%)	26 (81.3%)	
Elevated	21 (36.2%)	83 (24.6%)		10	40		2 (20.0%)	6 (18.7%)	
Unknown	0	0		0	0		4	9	

### Clinical Model

Multivariate logistic regression analysis identified location-L/L+D [β = -0.791, OR = 0.453 (95% CI, 0.247–0.832), *P* = 0.010] and Borrmann type [β = -1.132, OR = 3.103 (95% CI, 1.504–6.401), *P* = 0.002] as independent predictors of OPM status. A clinical model incorporating the location-L/L+D and the Borrmann type was developed.

### Annotation Type Analysis

**Figure 3:**
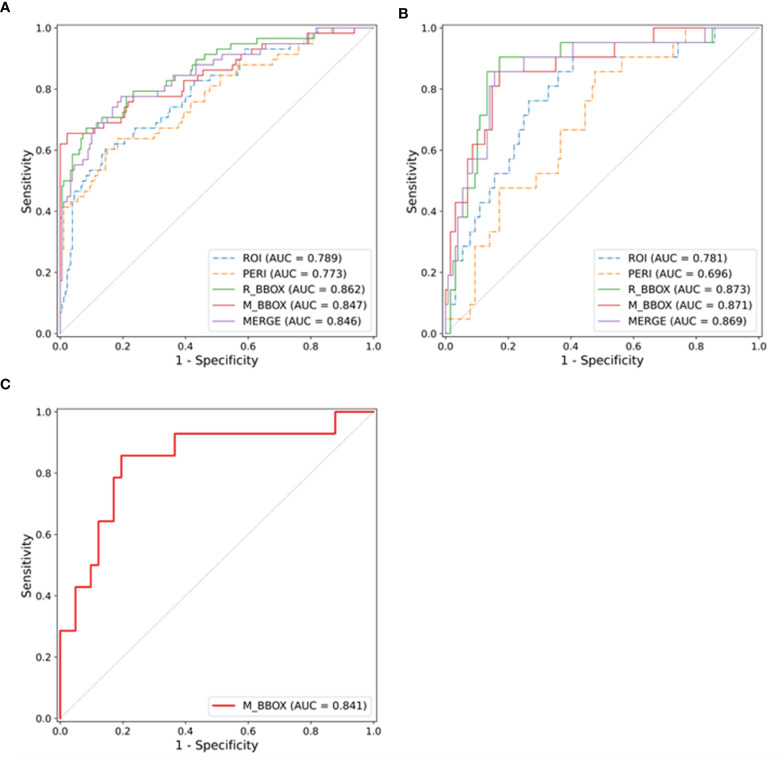
ROC curves of the radiomics models in the training cohort **(A)**, validation cohort **(B)** and external test cohort **(C)**, respectively.

We evaluated the impact of different annotation types on the performance of the radiomics model in the validation cohort. As shown in **Table 2**, after the three-step feature selection process, the number of radiomics features for the different annotation types was 3, 3, 2, 3 and 6. The radiomics model based on R_BBOX yielded an AUC of 0.873 (95% CI, 0.820–0.940), which was significantly better than the ROI model [AUC: 0.781 (95% CI, 0.710–0.863); *p* = 0.047]. Similarly, in the radiomics model that combined the tumor area and the PERI for analysis, an improvement in the predictive performance was noted [AUC: M_BBOX 0.871 (95% CI, 0.814–0.940), MERGE 0.869 (95% CI, 0.811–0.938)], although there was no significant difference by the Delong test (*p*: M_BBOX 0.081, MERGE 0.080). On the other hand, for the radiomics model that used only PERI for analysis, a decrease in the predictive performance was observed, with an AUC of 0.696 (95% CI, 0.620–0.785). After setting the threshold, the specificity of each model was compared when the sensitivity reached 0.8. The R_BBOX radiomics model produced the highest specificity, and the specificity of all radiomics models that combined tumor area and PERI exceeded 0.85 (specificity: R_BBOX 0.867, M_BBOX 0.852, MERGE 0.859). In contrast, the radiomics models using only tumor area or PERI had lower specificity (specificity: ROI 0.672, PERI 0.531). The ROC curves of the different radiomics models are shown in **Figure 3**.

**Table 2:** Performance of the radiomics models with different annotation types.

Annotation type	Feature count	AUC (95% CI)	Sensitivity	Specificity	*p*	p*
**ROI**	3	0.781 (0.710–0.863)	0.810	0.672	\	0.047
**PERI**	3	0.696 (0.620–0.785)	0.810	0.531	0.158	<0.001
**R_BBOX**	2	0.873 (0.820–0.940)	0.810	0.867	0.047	\
**M_BBOX**	3	0.871 (0.814–0.940)	0.810	0.852	0.081	0.937
**MERGE**	6	0.869 (0.811–0.938)	0.810	0.859	0.080	0.814

p is the Delong test result for ROIs versus other annotation types.

p* is the Delong test result for BBOX versus other annotation types.

ROI, region of interest; PERI, peritumoral area; R_BBOX, bounding box was obtained by calculating the minimum circumscribed rectangle of the ROI; M_BBOX, manual bounding box; AUC, area under the curve; CI, confidence interval.

M_BBOX was used as the final radiomics model because of its high prediction accuracy and extremely low annotation cost. The prediction score of the M_BBOX radiomics model was calculated as follows: -1.8863 + (-0.3683 * GLRLM_RunLengthNonUniformityNormalized) + (-0.0416 * GLDM_GrayLevelNonUniformity) + (-0.5014 * Shape2D_PerimeterSurfaceRatio).

### Comparison of the Clinical Model With the Radiomics Model

The comparison of the discrimination performance of the radiomics model and the clinical model in the validation cohort is shown in **Section 5** and **Table S2** in the **Supplementary Material**. The DeLong test showed that the diagnostic performance of the radiomics model was significantly better than that of the clinical model (*p* = 0.007). Both the sensitivity and specificity values of the radiomics model were higher than those of the clinical model (sensitivity: 0.857 *vs.* 0.524; specificity: 0.828 *vs.* 0.719).

As shown in **Section 5** and **Table S3** in the **Supplementary Material**, in the testing cohort, the AUC, sensitivity and specificity values of the radiomics model for all patients were 0.841 (95% CI, 0.697–0.956), 0.857 and 0.805, respectively. Considering that the clinical characteristics of some patients in the testing cohort were incomplete, a subset of the testing cohort containing 24 patients with complete clinical information was constructed. The predictive performances in the testing cohort (subset) are described in **Section 5** and **Table S3** in the **Supplementary Material**. Although there was no significant difference by the Delong test, the AUC of the radiomics model was better than that of the clinical model (AUC: radiomics model 0.889, clinical model 0.648), and it had higher specificity under the same sensitivity (specificity: radiomics model 0.778, clinical model 0.333).

### Clinical Use

Decision curve analysis (DCA) was used to evaluate the benefits of the radiomics model and both the all-laparoscopy and no-laparoscopy schemes. The net benefit of the no-laparoscopy scheme was always 0. The all-laparoscopy scheme means that additional laparoscopy was performed on all patients. The DCA results for the validation cohort are shown in **Figure S2A**. The radiomics model had the highest net benefit when the threshold probability in clinical decision-making was between 5% and 45%. **Figure S2B** shows the DCA results for the testing cohort, which indicated that the radiomics model had the highest net benefit when the threshold probability was between 10% and 60%.

### Four Sizes of Input Boxes

Our results indicated that the model achieved the highest AUC by using BBOX as input. (**Figures S2, S3**). However, when the size of the BBOX increased by 10 mm, 15 mm, or no limit, the performance of the models worsened, which might be because the larger the BBOX is, the more tissues are covered.

## Discussion

In this multicenter study, we developed a radiomics model based on 2D images annotated according to the BBOX containing the primary tumor and PERI to identify OPM in AGC patients prior to surgical treatment. The BBOX radiomics model had high diagnostic performance in the validation cohort, testing cohort (all), and testing cohort (subset).

The BBOX, an easy-to-use tool, can reduce the need for additional labeling and has been used in many clinical investigations (24–28). The region of the BBOX containing both the primary tumor and nearby peritoneum is larger than the ROI of the primary tumor. Recent reports (26, 29–32, 39, 40) have illustrated that peritumoral tissue-based radiomics analysis may reveal valuable information for diagnosis, prognosis and treatment response evaluations. Therefore, in our study, we focused on both the characteristic features of the primary tumor and the peritumoral tissue delineated with the BBOX to develop a radiomics model for the noninvasive diagnosis of OPM.

The analysis of peritumoral tissues surrounding the tumor mass can reveal important information related to tumor aggressiveness; it can reflect lymphovascular invasion, lymphangiogenesis, and angiogenesis (41–43) and provide other information that can be used for diagnostic and prognostic predictions (26, 29–32, 39–42). Moreover, such information may be effectively captured by radiomics analysis (26, 29–32, 39, 40). In our study, the nonoverlapping area between the tumor and R_BBOX was regarded as the PERI, which is theoretically a part of the whole peritumoral region. Our results indicated that the predictive value of PM based on the peritumoral region alone was limited, worse than that of the primary tumor alone, but showed significant improvement when integrated with the ROI of the primary tumor. This may be because the primary tumor, the main disease component, theoretically holds more information about tumor phenotypes than that of PERI, and BBOX covers more regions than the mass or peritoneal region alone.

Few studies (23–28) have investigated radiomic applications for PM diagnosis using CT. Liu et al. (21) and Kim et al. (22) focused on the preoperative CT texture features of the primary tumor area and the omentum area, respectively, to evaluate the possibility of PM in AGCs instead of considering the primary tumor and the surrounding tissue. Dong et al. (23) conducted a radiomics study using the CT phenotypes of primary tumors and the nearby peritoneum to accurately predict OPM in AGC patients. In their study, the diagnostic value of either the peritoneum or the mass was worse than the merged value for the detection of PM, which is consistent with our conclusions. However, they chose regions near the peritoneum rather than the primary tumor, which may be relatively far away from the mass. The delineated ROI of the peritoneum was part of the peritumoral tissue, and the annotated peritoneal area was >2 cm (2), which might not be representative of the entire surrounding condition of the mass on 2D CT images. In addition, manual sketching is a time-consuming and laborious process. The peritoneum is a wide and irregular structure without clear boundaries and forms multiple reflex structures (such as ligaments). Therefore, it is impossible to outline the whole peritoneum. This previous study also selected the local peritoneum, a small area. Although the researchers provided a rationale for selecting this method, delineating the peritoneal area of interest is still very subjective, and thus, it is difficult to determine reproducibility. We used the BBOX method to include all the information on the primary lesion at the 2D level and the information on the peritoneal area surrounding the lesion. These peritoneal areas were not selected subjectively; rather, they were selected incidentally based on their location relative to the primary lesion. Therefore, we believe that our delineation model is simple, easy to use, and reproducible.

Our results showed that by merging the ROI with the PERI, the radiomics model could achieve a higher AUC. This presented a question: what BBOX size would achieve satisfactory diagnostic value for identifying OPM? When the size of the BBOX increased by 10 mm, 15 mm, or no limit, the performance of the models worsened, which might be because the larger the BBOX is, the more tissues are covered. The tissues far away from the carcinoma provided less or no information about the neoplasms. Considering the lower annotation cost and similar AUC of the M_BBOX model compared with the R_BBOX model, the M_BBOX radiomics model was ultimately selected as the final radiomics model.

Laparoscopy remains a useful procedure for evaluating PM status (44–46), but it is invasive and expensive, and its specific applications for GC remain controversial. Our results revealed that the clinical model could not accurately predict PM status. The value of the radiomics model was then assessed by DCA. If the predicted probability of OPM is between 10 and 45%, more cases of undetected OPM on conventional CT images can be detected by the radiomics model than by the all-laparoscopy or no-laparoscopy scheme, avoiding unnecessary surgical procedures and extra costs. Furthermore, patients deemed to have a high possibility of OPM by the radiomics model may undergo diagnostic laparoscopy for further identification, helping to guide proper treatment.

## Limitations

There are several limitations to this study. First, we focused on 2D slices of lesions rather than whole lesions. In future work, automatic segmentation of the whole tumor is worth developing to better predict OPM status. Second, our study was retrospective in nature, and some initially available clinical factors were analyzed. Third, a small number of patients from two external centers were used to evaluate the generalizability of the radiomics model; however, large independent patient cohorts are still needed to validate our results.

## Conclusions

The radiomics model based on M_BBOX had higher diagnostic performance for the preoperative detection of OPM in AGC than the radiomics models based on tumor tissue or peritumoral tissue alone. In addition, its annotation is simpler and less time consuming.

## Data Availability Statement

The raw data supporting the conclusions of this article will be made available by the authors, without undue reservation.

## Ethics Statement

The studies involving human participants were reviewed and approved by Ethics Committee on Biomedical Research, West China Hospital of Sichuan University. The patients/participants provided their written informed consent to participate in this study.

## Author Contributions

DL: data curation, software, formal analysis, validation, investigation, methodology, and writing original draft. WZ: data curation, formal analysis, validation, investigation, methodology, and writing original draft. FH: resources, data curation, software, formal analysis, validation, investigation, and writing original draft. PY: data curation, software, formal analysis, validation, investigation, methodology, and visualization. XZ: resources, data curation, software, formal analysis, and validation. HY: conceptualization, data curation, software, formal analysis, validation, and methodology. LY: data curation, formal analysis, and investigation. XF: data curation, formal analysis, and investigation. BS: resources, data curation, formal analysis, supervision, and methodology. BW: resources, data curation, formal analysis, supervision, and methodology. JH: conceptualization, resources, data curation, formal analysis, supervision, funding acquisition, validation, investigation, methodology, project administration, and writing review and editing. ZH: conceptualization, resources, data curation, formal analysis, supervision, validation, investigation, methodology, writing original draft, project administration, and writing review and editing. All authors contributed to the article and approved the submitted version.

## Funding

This work was supported by the 1*3*5 Project for Disciplines of Excellence, West China Hospital, Sichuan University (No. ZY2017304).

## Conflict of Interest

Authors PY and HY were employed by company Infervision.

The remaining authors declare that the research was conducted in the absence of any commercial or financial relationships that could be construed as a potential conflict of interest.

## Publisher’s Note

All claims expressed in this article are solely those of the authors and do not necessarily represent those of their affiliated organizations, or those of the publisher, the editors and the reviewers. Any product that may be evaluated in this article, or claim that may be made by its manufacturer, is not guaranteed or endorsed by the publisher.
