# Supplementary material for: A Bounding Box-Based Radiomics Model for Detecting Occult Peritoneal Metastasis in Advanced Gastric Cancer: A Multicenter Study
Source: Front Oncol. 2021 Dec 3;11:777760. doi: 10.3389/fonc.2021.777760 (PMC8678129; doi:10.3389/fonc.2021.777760)
Supplement: Supplementary file 1 [file DataSheet_1.pdf]

Supplementary Material for
"A Bounding Box-Based Radiomic Model for Detecting Occult Peritoneal Metastasis in Advanced Gastric Cancer: A Multicenter Study"

Section 1: PM status confirmation

All patients from three centers were initially diagnosed as OPM-negative according to the vein-phase CT images, and were confirmed to have peritoneal metastasis (PM) by surgical or laparoscopic exploration. The laparoscopy procedure used here was a four-step procedure for laparoscopic exploration in GC patients[1]. During the procedure, the abdominal and peritoneal conditions were carefully examined, and all suspicious peritoneal implants or ascites were sent for pathological biopsy or cytological confirmation. The existence of PM was determined using the American Joint Committee on Cancer guidelines in consensus by pathologists and surgeons.

Section 2: Patient recruitment

The patient inclusion criteria were:

(1) Primary gastric adenocarcinoma diagnosed by endoscopy-biopsy pathology and advanced gastric cancer (AGC) (cT $\geq$ 2) diagnosed by endoscopy-biopsy and CT; (2) venous images of the whole abdomen (with a slice thickness of 2 mm) were obtained preoperatively, and later laparoscopy or surgery was performed within 2 weeks; (3) no typical PM findings, such as omental nodules or omental cake, extensive ascites, or irregular thickening with high peritoneal enhancement, on CT; and (4) no indications of distant metastasis or other tumors.

The exclusion criteria were as follows:

(1) Previous abdominal surgery; (2) previous abdominal malignancies or inflammatory diseases; (3) insufficient distention of the stomach; (4) poor imaging quality due to artifacts; and (5) indiscernible primary GC tumor on CT images.

This multicenter study design is shown in Figure S1. A total of 599 patients from three centers were enrolled; 93 were confirmed as OPM-positive and 506 were confirmed as OPM-negative. A total of 544 patients meeting the inclusion/exclusion criteria from West China Hospital were divided into a training cohort (58 occult peritoneal metastasis (OPM)-positive patients and 337 OPM-negative patients) and a validation cohort (21 OPM-positive patients and 128 OPM-negative patients). Twenty-two patients from People's Hospital of Leshan between November 2017 and June 2018 met the inclusion/exclusion criteria; these patients included 4 OPM-negative patients and 17 OPM-positive patients. Thirty-eight patients (10 OPM-negative and 28 OPM-positive patients) from The First Affiliated Hospital of Chengdu Medical College in China between January 2018 and July 2020 were enrolled in this study.

Section 3: CT examinations

Prior to the CT examination, patients were requested to fast from food for at least 6 h and limit water intake to 600 – 1000 mL to achieve gastric distension prior to the examination. Patients were first trained to hold their breath during CT scanning. The scan covered the entire abdomen. The parameters of the CT protocol are listed in Table S1.

Section 4: Feature selection

The feature selection process included three steps as shown in the Section 4 and aimed to avoid overfitting during the model-building process and potential biases associated with the results. The first step was to perform a Mann-Whitney U test between OPM-positive and OPM-negative patients for all features and sort the results by p value from largest to smallest. Accordingly, the significance threshold was set to 0.05 to include only the significant features for the following process. The second step was to

calculate the Pearson correlation coefficient between each pair of retained features and define this value as r. If there was a pair of features with $|r| > 0.80$, the one with a larger p value calculated in the first step was excluded. The last step was a model-based feature selection method called Boruta[2]. The goal of Boruta was to rank all the features related to OPM positivity by relevance.

Section 5: The performance evaluation of the radiomics and clinical model

The performance evaluation of the radiomics and clinical model in the validation cohort and the testing cohort are listed in Table S2 and Table S3, respectively .

Section 6: Annotation error analysis

Taking into account the possible errors of manual bounding box annotation, a simulation experiment was conducted in this study. This experiment only included data from the training cohort and validation cohort and followed the same data division process. Four levels of annotation errors were set in this study: 5 mm, 10 mm, 15 mm, and unlimited. The annotation error of a BBOX refers to the maximal extent to which any side of the BBOX could expand outward. Under each level of annotation error, 100 different random BBOXes were generated from all data, called range5_BBOX, range10_BBOX, range15_BBOX and unlimited_BBOX. As annotation error increased, the maximal area covered by annotation also increased, as shown in Figure S3.

For each error level, we conducted 1000 experiments, and the experimental procedure was as follows: (1) For each patient, we randomly selected one of 100 annotations as the annotation result. (2) We extracted the radiomic features of each patient from the selected annotations. (3) In the training cohort, the three-step feature selection process was applied to select the radiomics features and build the radiomics model. (4) We evaluated the predictive performance of the radiomics model in the validation cohort.

The model performance distribution of experiments at each annotation error level is shown in Figure S4. The median and interquartile points of the area under the curve (AUC) values for the range5_BBOX radiomics models, range10_BBOX radiomics models, range15_BBOX radiomics models and unlimited_BBOX radiomics models were denoted as 0.859 (95% CI, 0.841-0.867), 0.849 (95% CI, 0.837-856), 0.842 (95% CI, 0.825-0.853) and 0.725 (95% CI, 0.654-0.780), respectively.

References

1. Liu K, Chen XZ, Zhang WH, et al. "Four-Step Procedure" of laparoscopic exploration for gastric cancer in West China Hospital: a retrospective observational analysis from a high-volume institution in China. *Surg Endosc.* 2019;33(5):1674-1682.

2. Kursa MB RW. Feature selection with the boruta package. *J Stat Softw.* 2010;36:1-13.

Supplementary Tables

Table S1. The CT protocols of the three centers

Parameter	Center 1	Center 2	Center 3
**CT scanner**	Siemens Somatom Definition AS+, Siemens Somatom Definition	Siemens Somatom Definition AS +	Philips Brilliance 64
Tube voltage (KV)	120	120	120
Amperage (mAs)	210	210	110-170
Slice thickness (mm)	2	2	2
Slice interval (mm)	2	2	2
Field of view (cm)	35~50	35~50	35
Image matrix	512 × 512	512 × 512	512 × 512
rotation time (s)	0.5	\	0.5
Pitch	1.0	0.6	0.891
**Contrast agent**	Iopamiro, 370 mg I/mL	\	Ioversol, 300 mg I/mL
**Contrast agent infused Rate** (mL/s)	2.5 – 3.0	3.0	3.0
**Contrast agent infused dosage** (mL/kg)	1.2 – 1.5	1.2 – 1.5	1.2 – 1.5
**Portal vein phase CT images**	The precontrast phase, the arterial phase at the trigger, and the portal vein phase 30 s after the trigger were obtained with a trigger threshold of 170 HU in the aorta.	The precontrast phase, the arterial phase at the trigger, and the portal vein phase 30 s after the trigger were obtained with a trigger threshold of 170 HU in the aorta.	The precontrast phase, the arterial phase at the trigger, and the portal vein phase 30 s after the trigger were obtained with a trigger threshold of 170 HU in the aorta.

Table S2. Performance evaluation of the radiomics and clinical model in the validation cohort.

Performance	Validation cohort	
	Radiomics Model	Clinical Model
**TP**	18	11
**TN**	106	92
**FN**	3	10
**FP**	22	36
**Sensitivity**	0.857	0.524
**Specificity**	0.828	0.719
**AUC (95% CI)**	0.871 (0.814-0.940)	0.670 (0.615-0.739)
p	0.007	\

Abbreviations: True positive, TP; True negative, TN; False negative, FN; False positive, FP; Area under the curve, AUC.

Table S3. Performance evaluation of the radiomics and clinical model in the testing cohort.

Performance	Testing cohort (All)	Testing cohort (subset)	
	Radiomics Model	Radiomics Model	Clinical Model
**TP**	12	5	5
**TN**	33	14	6
**FN**	2	1	1
**FP**	8	4	12
**Sensitivity**	0.857	0.833	0.833
**Specificity**	0.805	0.778	0.333
**AUC (95% CI)**	0.841 (0.697-0.956)	0.889 (0.713-1.000)	0.648 (0.427-0.859)
p	\	0.167	\

Abbreviations: True positive, TP; True negative, TN; False negative, FN; False positive, FP; Area under the curve, AUC.

Supplementary Figure Legend

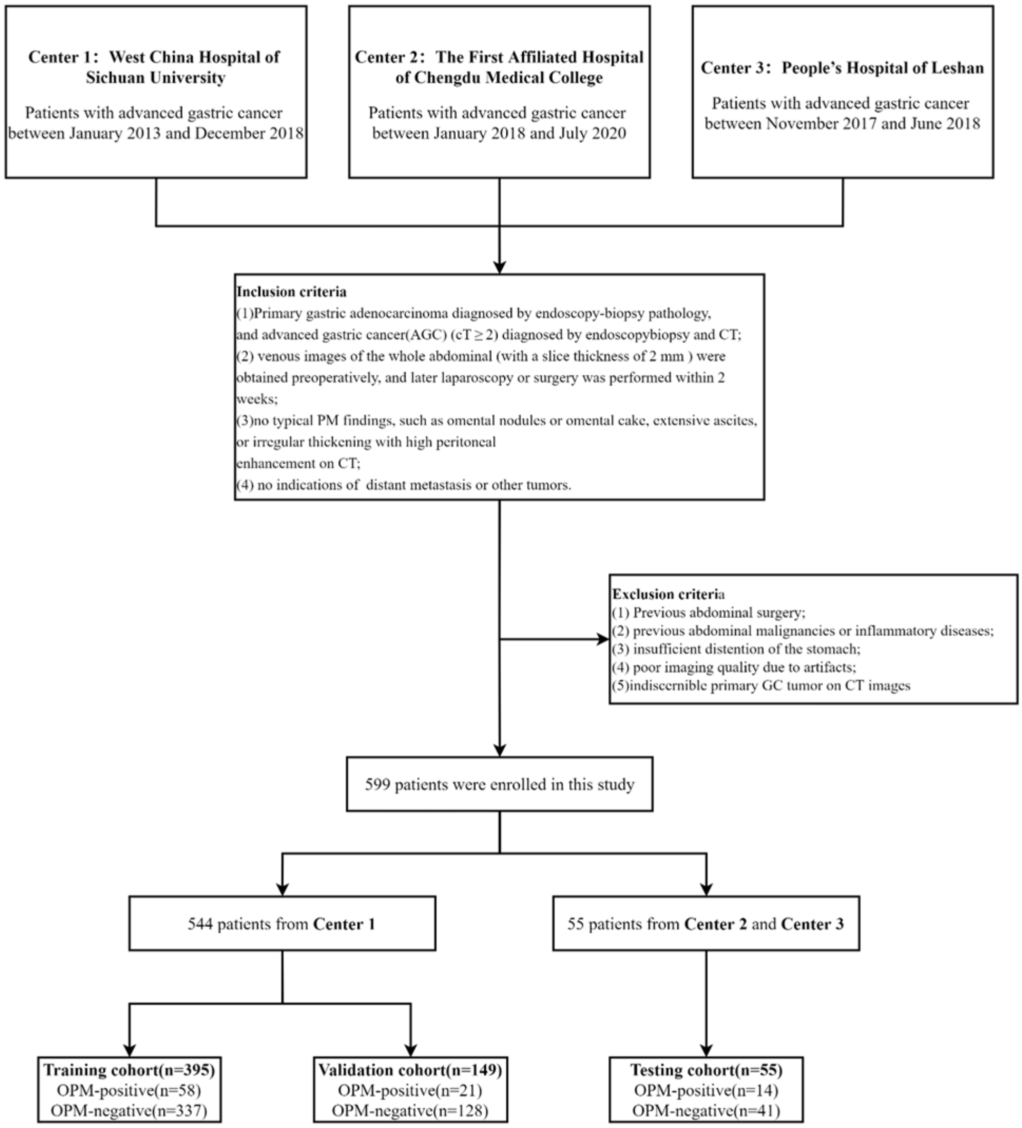

Figure S1. The Flow diagram of study population

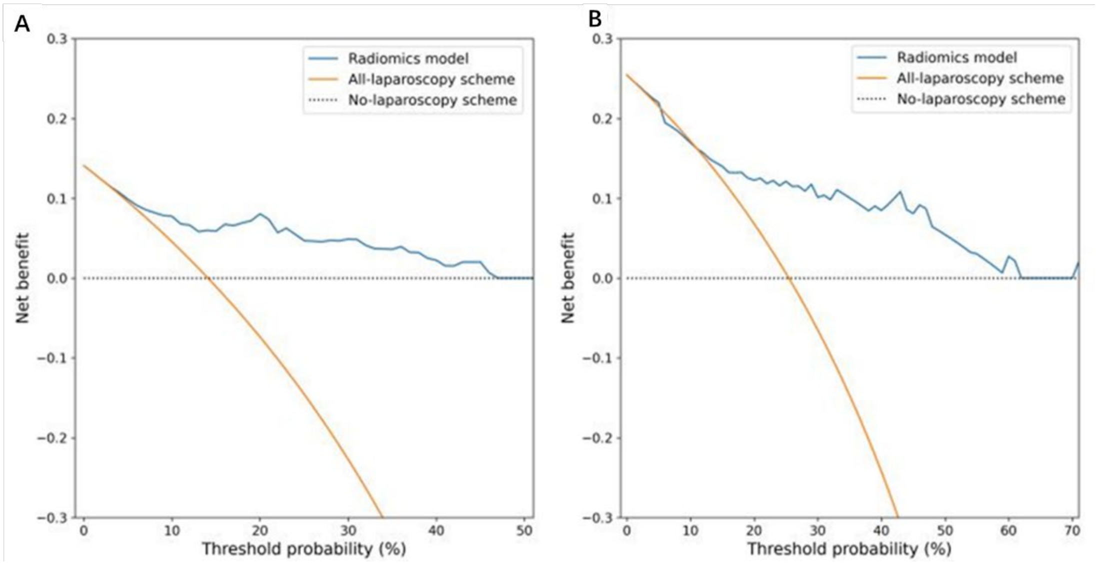

Figure S2. DCA for the radiomics model, the All-laparoscopy scheme and the No-laparoscopy scheme. Blue line: the Radiomics model. Orange line: the All-laparoscopy scheme, assuming that all patients should undergo laparoscopy to confirm the presence of OPM. Dotted line: the No-laparoscopy scheme, assuming no presence of OPM in patients (i.e., the premise of OPM).

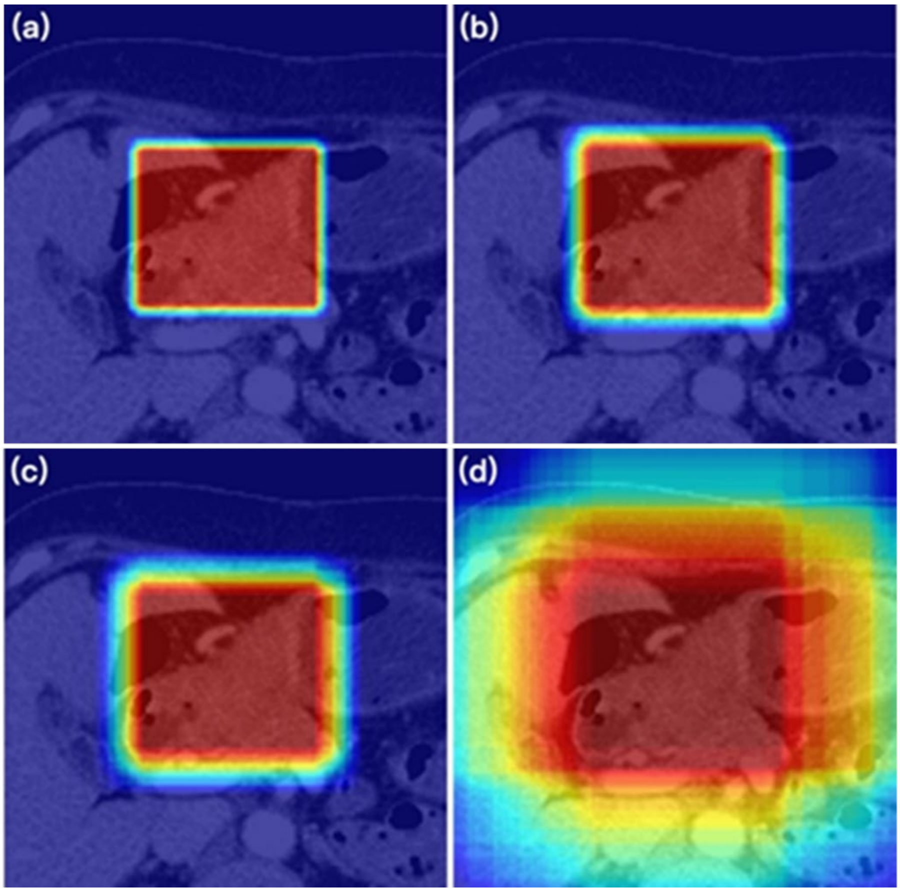

Figure S3. Areas covered by 100 random BBOXes. (a)-(d), range5_BBOX, range10_BBOX, range15_BBOX and unlimited_BBOX models. Red indicates the area covered by all BBOXes with a

probability of 1 in red and a probability of 0 in blue; the colors in between indicate a decreasing probability as the color approaches blue.

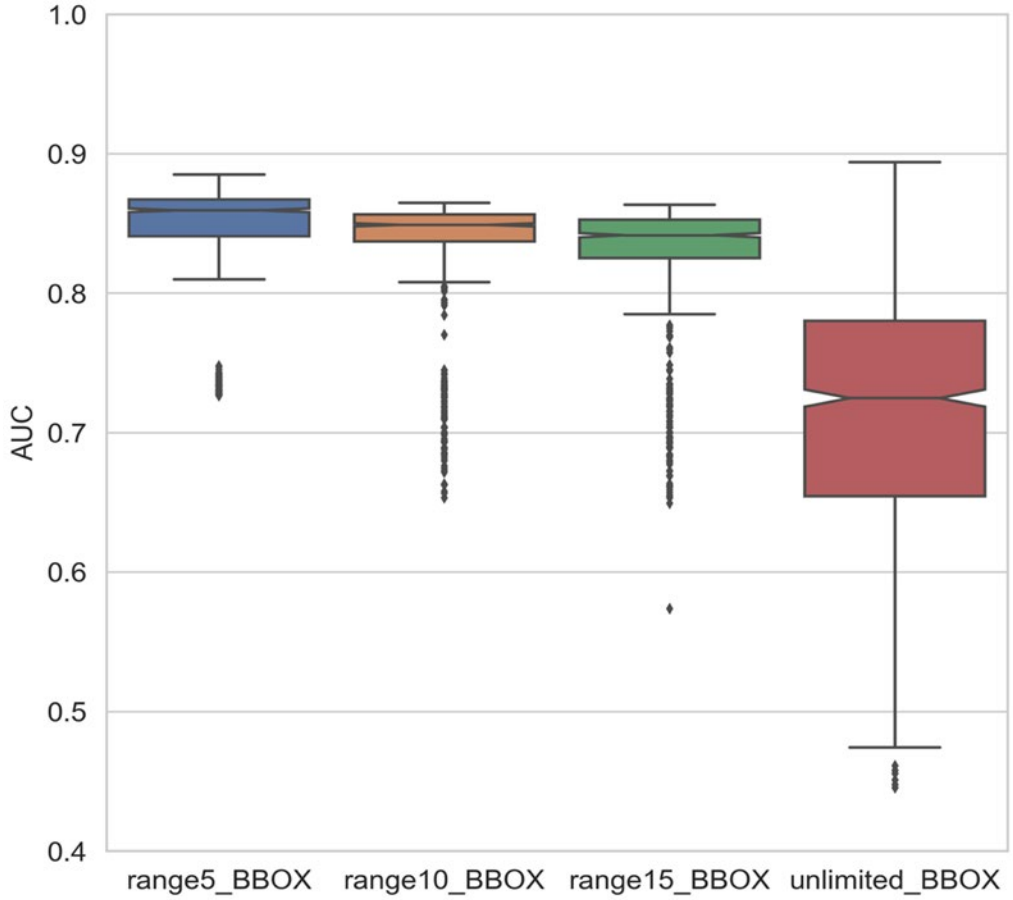

Figure S4. Boxplots for AUCs by annotation error levels

Supplementary IBSI reporting

Image processing - image interpolation	For the image, we use the trilinear interpolation algorithm to unify the in-plane resolution of the image to 1mm x 1mm.<br>For the mask, we use the nearest neighbor interpolation algorithm for resolution adjustment.<br>We use the simpleITK package in python to execute the interpolation algorithm. For details, please refer to the official documentation of the simpleITK package.
Image processing – discretization	We use the pyradiomics package in python to perform radiomics feature extraction, and the discretization process is built into the extraction process. We use a fixed bin size for discretization, the width of the bin is 32, and the starting value is -1000.
Image processing - Filter	We didn’t transform the image, so we didn’t use any filters.
Feature cluster	**Shape (n = 9)**<br>'Elongation', 'MajorAxisLength', 'MaximumDiameter', 'MeshSurface', 'MinorAxisLength', 'Perimeter', 'PerimeterSurfaceRatio', 'PixelSurface', 'Sphericity'<br><br>**Firstorder (n=18)**<br>'10Percentile', '90Percentile', 'CoefficientOfVariation', 'Energy', 'InterquartileRange', 'Kurtosis', 'Maximum', 'Mean', 'MeanAbsoluteDeviation', 'Median', 'MedianAbsoluteDeviation', 'Minimum', 'QuartileCoefficientOfDispersion', 'Range', 'RobustMeanAbsoluteDeviation', 'RootMeanSquared', 'Skewness', 'Variance'<br><br>**Histogram (n=18)**<br>'10Percentile', '90Percentile', 'CoefficientOfVariation', 'Entropy', 'InterquartileRange', 'Kurtosis', 'Maximum', 'Mean', 'MeanAbsoluteDeviation', 'Median', 'MedianAbsoluteDeviation', 'Minimum', 'QuartileCoefficientOfDispersion', 'Range', 'RobustMeanAbsoluteDeviation', 'Skewness', 'Uniformity', 'Variance'<br><br>**GLDM (n=14)**<br>'DependenceEntropy', 'DependenceNonUniformity', 'DependenceNonUniformityNormalized', 'DependenceVariance', 'GrayLevelNonUniformity', 'GrayLevelVariance', 'HighGrayLevelEmphasis', 'LargeDependenceEmphasis', 'LargeDependenceHighGrayLevelEmphasis', 'LargeDependenceLowGrayLevelEmphasis', 'LowGrayLevelEmphasis', 'SmallDependenceEmphasis', 'SmallDependenceHighGrayLevelEmphasis', 'SmallDependenceLowGrayLevelEmphasis'<br><br>**GLCM (n=23)**<br>'Autocorrelation', 'ClusterProminence', 'ClusterShade', 'ClusterTendency', 'Contrast', 'Correlation', 'DifferenceAverage', 'DifferenceEntropy', 'DifferenceVariance', 'Id', 'Idm', 'Idmn', 'Idn', 'Imc1', 'Imc2', 'InverseVariance', 'JointAverage', 'JointEnergy', 'JointEntropy', 'MCC', 'MaximumProbability', 'SumEntropy', 'SumSquares'<br><br>**GLSZM (n=16)**<br>'GrayLevelNonUniformity', 'GrayLevelNonUniformityNormalized', 'GrayLevelVariance', 'HighGrayLevelZoneEmphasis', 'LargeAreaEmphasis', 'LargeAreaHighGrayLevelEmphasis', 'LargeAreaLowGrayLevelEmphasis', 'LowGrayLevelZoneEmphasis', 'SizeZoneNonUniformity', 'SizeZoneNonUniformityNormalized', 'SmallAreaEmphasis', 'SmallAreaHighGrayLevelEmphasis', 'SmallAreaLowGrayLevelEmphasis', 'ZoneEntropy', 'ZonePercentage', 'ZoneVariance'<br><br>**GLRLM (n=16)**

	'GrayLevelNonUniformity', 'GrayLevelNonUniformityNormalized', 'GrayLevelVariance', 'HighGrayLevelRunEmphasis', 'LongRunEmphasis', 'LongRunHighGrayLevelEmphasis', 'LongRunLowGrayLevelEmphasis', 'LowGrayLevelRunEmphasis', 'RunEntropy', 'RunLengthNonUniformity', 'RunLengthNonUniformityNormalized', 'RunPercentage', 'RunVariance', 'ShortRunEmphasis', 'ShortRunHighGrayLevelEmphasis', 'ShortRunLowGrayLevelEmphasis'<br><br>**NGTDM (n=5)**<br>'Busyness', 'Coarseness', 'Complexity', 'Contrast', 'Strength'
